# Knowledge, Attitudes, and Practices Associated with Chronic Kidney Disease in Northern Tanzania: A Community-Based Study

**DOI:** 10.1371/journal.pone.0156336

**Published:** 2016-06-09

**Authors:** John W. Stanifer, Elizabeth L. Turner, Joseph R. Egger, Nathan Thielman, Francis Karia, Venance Maro, Kajiru Kilonzo, Uptal D. Patel, Karen Yeates

**Affiliations:** 1 Department of Medicine, Duke University, DUMC Box 3182, Durham, NC, 27710, United States of America; 2 Duke Global Health Institute, Duke University, Durham, NC, 27710, United States of America; 3 Duke Clinical Research Institute, Duke University, DUMC Box 3646, Durham, NC, 27710, United States of America; 4 Department of Biostatistics and Bioinformatics, Duke University, DUMC Box 2721, Durham, NC, 27710, United States of America; 5 Kilimanjaro Christian Medical College, Sokoine Road, Moshi, Tanzania; 6 Department of Medicine, Queen’s University, 76 Stuart Street, Kingston, Ontario, Canada K7L 2VL; RTI International, UNITED STATES

## Abstract

**Background:**

Non-communicable diseases (NCDs) are a leading cause of death among adults in sub-Saharan Africa, and chronic kidney disease (CKD) is a growing public health threat. Understanding knowledge, attitudes, and practices associated with NCDs is vital to informing optimal policy and public health responses in the region, but few community-based assessments have been performed for CKD. To address this gap, we conducted a cross-sectional survey of adults in northern Tanzania using a validated instrument.

**Methods:**

Between January and June 2014, we administered a structured survey to a random sample of adults from urban and rural communities. The validated instrument consisted of 25 items designed to measure knowledge, attitudes, and practices associated with kidney disease. Participants were also screened for CKD, diabetes, hypertension, and human immunodeficiency virus.

**Results:**

We enrolled 606 participants from 431 urban and rural households. Knowledge of the etiologies, symptoms, and treatments for kidney disease was low (mean score 3.28 out of 10; 95% CI 2.94, 3.63). There were no significant differences by CKD status. Living in an urban setting and level of education had the strongest independent associations with knowledge score. Attitudes were characterized by frequent concern about the health (27.3%; 20.2, 36.0%), economic (73.1%; 68.2, 77.5%), and social impact (25.4%; 18.6, 33.6%) of kidney disease. Practices included the use of traditional healers (15.2%; 9.1, 24.5%) and traditional medicines (33.8%; 25.0, 43.9%) for treatment of kidney disease as well as a willingness to engage with mobile-phone technology in CKD care (94.3%; 90.1, 96.8%).

**Conclusions:**

Community-based adults in northern Tanzania have limited knowledge of kidney disease. However, there is a modest knowledge base upon which to build public health programs to expand awareness and understanding of CKD, but these programs must also consider the variety of means by which adults in this population meet their healthcare needs. Finally, our assessment of local attitudes suggested that such public health efforts would be well-received.

## Introduction

In sub-Saharan Africa, non-communicable diseases (NCDs) are now one of the leading causes of death among adults, and chronic kidney disease (CKD) is being recognized as a NCD with high prevalence, morbidity, and mortality [1–3]. In parts of Tanzania, for example, the prevalence of CKD has been estimated at 7% with as many as 15% of adults in urban settings living with CKD. However, despite a potentially high prevalence there remains a dearth of epidemiological data regarding disease burden and risk factors, and community awareness of this condition remains low [1, 4]. In addition to the need for more epidemiological assessments, there is an urgent need for a better understanding of knowledge, attitudes, and practices associated with NCDs such as CKD. These factors can provide important insights that inform optimal policy and public health responses.

Few community-based studies have assessed knowledge, attitudes, and practices associated with kidney disease in sub-Saharan Africa; none to our knowledge have done so in East Africa. The Comprehensive Kidney Disease Assessment for Risk Factors, epidemiology, Knowledge, and Attitudes (CKD-AFRiKA) study is an ongoing project in northern Tanzania with the aim of understanding the epidemiology, etiology, knowledge, attitudes, and practices associated with CKD especially as it relates to other NCDs.

As part of the CKD AFRiKA study, we conducted a community-based cross sectional survey in which we administered a validated survey to a representative sample of Swahili-speaking adults [5]. The current study sought to describe the knowledge, attitudes, and practices (KAP) related to kidney disease among a community-based sample of adults, and we sought to characterize the demographic determinants of these attributes. A primary aim of the study was to assess potential differences in the distribution of knowledge, attitudes, and practices among community members with CKD or at risk for CKD. A secondary aim was to explore potential differences in these attributes across several demographic characteristics including gender, age, education, ethnicity, and setting.

## Methods

### Ethics Statement

The study protocol was approved by Duke University Institutional Review Board (#Pro00040784), the Kilimanjaro Christian Medical College Ethics Committee (EC#502), and the National Institute for Medical Research in Tanzania. Written informed consent (by signature or thumbprint) was obtained from all participants. All participants with abnormal findings received counseling, educational pamphlets, and reimbursement with referral for follow-up.

### Study Setting

We conducted a stratified, cluster-designed cross-sectional survey between January and June 2014 in the Kilimanjaro Region of Tanzania. The region comprises seven districts. Our study was conducted in two of these districts, Moshi Urban and Moshi Rural, which served as strata for our sampling scheme. The adult regional population is more than 900,000 people of whom 35% live in an urban setting. The region has a slight female majority (58%). The unemployment rate is 19%, and the majority of adults have a primary education or less (77%). Regional literacy rates are greater than 80% for both men and women. The largest ethnic group is the Chagga tribe followed by the Pare, Sambaa, and Maasai tribes [6, 7]. All participants in our study spoke Swahili.

### Survey Instrument

The details of the creation and validation of the survey instrument have been described previously in detail [5]. In brief, we used a four-stage process that included the survey construction, translation, qualitative piloting, and demonstration of test-retest reliability and construct validity through the known-groups method [8]. The final version administered as part of this study consisted of 25 items divided into three conceptual domains: knowledge, attitudes, and practices (S1 Appendix). The knowledge domain was designed to test knowledge of the etiologies, diagnosis, and treatment of kidney disease as well as the normal function of the kidneys. It comprised ten items each measured with a four-point categorical response scale (‘Yes’, ‘No’, ‘Do Not Know’ and ‘Unsure’). The attitudes domain comprised eight items each measured on a dichotomous response scale (‘Yes’ and ‘No’). The practice domain was designed to test hypothetical practices associated with a diagnosis of kidney disease. It comprised seven items each measured on a four-point Likert-based scale (‘Very Unlikely’, ‘Unlikely’, ‘Likely’ and ‘Very Likely’).

### Data Collection

Using two local surveyors, we administered the KAP survey instrument using a three-stage cluster probability sampling method stratified by urban and rural status. Our sampling methods have been previously described in detail [4]. In brief, we used a random-number generator to select thirty seven neighborhoods based on probability proportional to size using the 2012 Tanzanian National Census [6]. Within each neighborhood a cluster site was determined using geographic points randomly generated using Arc Global Information Systems (ArcGIS), v10.2.2 (Environmental Systems Research Institute, Redlands, CA), and households were then randomly chosen based on coin-flip and die-rolling techniques according to a pre-established protocol. The sample size was based on the requirements of the CKD AFRIKA study which was designed to estimate the community prevalence of CKD with a precision of 5% when accounting for the cluster-design effect. All adults (age ≥ 18 years old) who were Tanzanian citizens and spoke Swahili were eligible for inclusion. To reduce non-response rates, we attempted a minimum of two additional, after- hours visits.

We collected demographic information for each participant, and we then administered the survey verbally in Swahili. Both surveyors were instructed to answer questions related to clarifying instructions and words, but they were instructed not to answer questions related to the meaning of each question. When possible, the instrument was administered in private.

### Disease Definitions

Following completion of the KAP survey, participants were offered free screening for CKD, diabetes, hypertension, and human immunodeficiency virus (HIV) as part of the CKD AFRIKA Study [4]. Chronic kidney disease was defined as the presence of albuminuria (≥30mg/dL confirmed by repeat assessment) and/or a once-measured estimated glomerular filtration rate (GFR) ≤60 ml/min/1.73m^2^ according to the Modification of Diet in Renal Disease equation without the race factor [9]. Hypertension was defined as a single blood pressure measurement of greater than 160/100 mmHg, a two-time average measurement of greater than 140/90 mmHg, or the ongoing use of anti-hypertensive medications. Diabetes was defined as an HbA1c level ≥ 7.0% or the ongoing use of anti-hyperglycemic medications. HIV was defined as a positive Alere Determine HIV -1/2 assay (Alere Medical Co. Ltd; Waltham, MA) confirmed by a Uni-Gold HIV assay (Trinity Biotech Manufacturing Ltd; Wicklow, Ireland), a self-reported history, or the ongoing use of highly active anti-retroviral therapy.

We considered participants who had poorly controlled diabetes, hypertension, or HIV or who lacked awareness of having diabetes, hypertension, or HIV as being at high risk for CKD. We defined poorly controlled diabetes as an HbA1c level ≥7.0% with or without therapy; poorly controlled hypertension as a two-time blood pressure of ≥140/90 mmHg or a one-time blood pressure >160/100mmHg with or without therapy; and poorly controlled HIV as being positive for HIV but not currently receiving biomedical care.

### Data Analysis

We used STATA version 13 (STATA Corp., College Station, TX) for all data analyses. Continuous variables are summarized by their mean and standard deviation (SD) and categorical variables as crude counts and percentages. Differences in un-weighted proportions between groups were compared using the chi-square test for independence or Fisher’s exact test. All p-values are two-sided.

For the knowledge domain, we calculated a composite score (range: 0–10). To do so, we first scored each item as correct (1) or incorrect (0) with the responses ‘Do Not Know’ and ‘Unsure’ treated as incorrect. We then obtained the sum of the 10 scored items. Composite scores are reported as mean (95% confidence interval [CI]) and were sample-balanced using age- and gender-weights based on the 2012 urban and rural district-level census data (6). For the attitudes domain, each item was scored as ‘Yes’ (1) or ‘No’ (0), and for the practices domain, each item was scored as ‘Very unlikely’ (0), ‘Unlikely’ (0), ‘Likely’ (1), or ‘Very likely’ (1). Summary estimates for these domains were also sample-balanced using age- and gender-weights based on the 2012 urban and rural district-level census data [6].

Crude and adjusted mean differences (MD) were estimated using generalized linear models assuming an identity link. We accounted for the design effect due to cluster sampling by using the Taylor Series linearization method implemented using the ‘svy suite’ of commands in STATA. For the knowledge domain, separate univariable models were fitted to mean knowledge score for each variable including gender, education, setting (urban or rural), occupation, age, ethnicity, self-reported history of NCD or HIV, CKD, and at risk for CKD. A multi-variable model was fitted to mean knowledge score for the independent variables associated with the outcome, including level of education, setting, age, occupation, and self-reported history of HIV. A matrix correlation among these five variables did not show evidence for strong (r >0.50) collinearity between any two variables. A backward stepwise procedure was used to specify the final model utilizing a modified Wald test to compare nested models.

## Results

**Fig 1:**
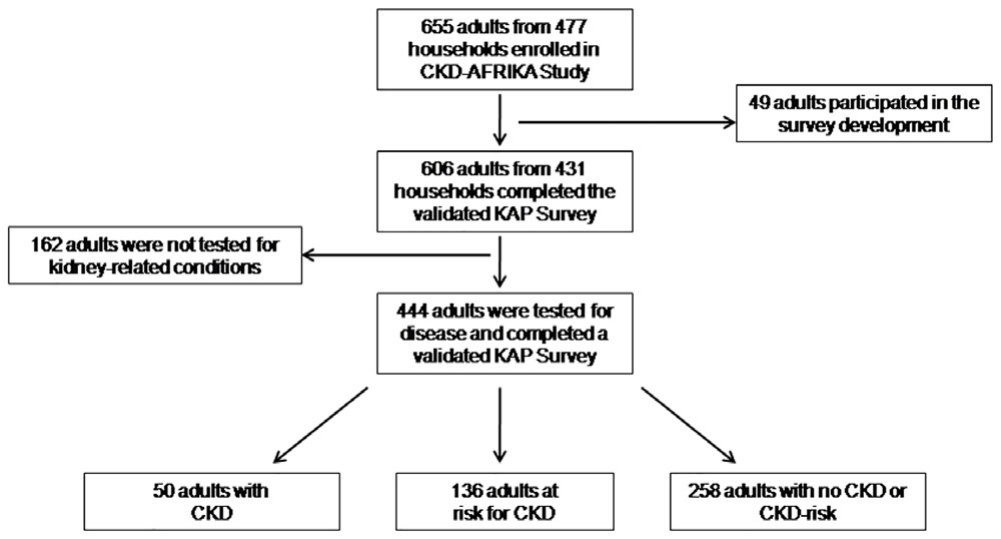
Flow diagram. Participants who were enrolled in the study; CKD-AFRiKA.

Between January and June 2014, we enrolled 655 adults from 477 households with a household non-response rate of 15% (Fig 1). As shown, 49 of these participated in the validation study, and the remaining 606 completed a validated KAP survey. From these, 444 were tested for a kidney-related condition (CKD, diabetes, hypertension, HIV).

Among the 606 participants who completed the validated KAP survey, the mean un-weighted age was 45.5 years (SD 15.5), 149 were men (24.6%), 463 (76.4%) lived in an urban setting, 363 (59.9%) were ethnically Chagga, 258 (39.4%) were farmers or daily wage-earners, and 451 (74.4%) had only a primary school education. (Table 1). Compared to participants without CKD but at risk, those with CKD were slightly younger, lived in an urban setting, were more frequently occupied as professional or small business owners, and had fewer co-morbidities.

**Table 1:** Demographic, social characteristics, self-reported medical history, and co-morbidities of the study population stratified by CKD risk status; N = 606 (CKD-AFRIKA, 2014).

Category	Variable	With CKD (n = 50)	At Risk for CKD (n = 136)	p-value^±^	Total (n = 606)
Demographics	**Gender (%)**			0.76	
	Male	17 (34.0%)	43 (31.6%)		149 (24.6)
	Female	33 (66.0%)	93 (68.4%)		457 (75.4)
	**Age (%)**			0.08	
	18–39 years old	15 (30.0%)	24 (17.7%)		248 (40.9)
	40–59 years old	21 (42.0%)	52 (38.2%)		225 (37.1)
	60+ years old	14 (28.0%)	60 (44.1%)		133 (22.0)
	**Ethnicity (%)**			0.76	
	Chagga	36 (72.0%)	95 (69.9%)		363 (59.9)
	Pare	5 (10.0%)	10 (7.4%)		79 (13.1)
	Sambaa	2 (4.0%)	4 (2.9%)		33 (5.5)
	Other^#^	7 (14.0%)	27 (19.9%)		131 (21.5)
	**Setting**			<0.01	
	Rural	3 (6.0%)	38 (27.9%)		143 (23.6%)
	Urban	47 (94.0%)	98 (72.1%)		463 (76.4%)
Socials Characteristics	**Education (%)**			0.35	
	None	3 (6.0%)	17 (12.5%)		29 (4.8)
	Primary	34 (68.0%)	92 (67.7%)		451 (74.4)
	Secondary	7 (14.0%)	19 (14.0%)		92 (15.2)
	Post-Secondary	6 (12.0%)	8 (5.9%)		34 (5.6)
	**Occupation (%)**			0.07	
	Unemployed	6 (12.0%)	15 (11.0%)		136 (20.8)
	Farmer/Wage Earner	13 (26.0%)	63 (46.3%)		258 (39.4)
	Small Business/Vendors	19 (38.0%)	32 (23.5%)		198 (30.2)
	Professional^†^	12 (24.0%)	26 (19.1%)		63 (9.6)
Self-Reported Medical History	**Non-Communicable Diseases (%)**				
	Diabetes	12 (24.0%)	26 (19.1%)	0.46	61 (10.1)
	Hypertension	18 (36.0%)	51 (37.5%)	0.85	143 (23.7)
	Stroke	2 (4.0%)	3 (2.2%)	0.50	7 (1.2)
	Heart Disease^‡^	2 (4.0%)	3 (2.2%)	0.51	14 (2.3)
	COPD	0 (0.00%)	1 (0.74%)	0.54	6 (1.0)
	Kidney Disease	5 (10.0%)	1 (0.74%)	<0.01	13 (2.2)
	**Communicable Diseases (%)**				
	Malaria	47 (94.0%)	115 (84.6%)	0.09	544 (89.8)
	Tuberculosis	0 (0.00%)	3 (2.2%)	0.29	11 (1.8)
	HIV/AIDS	6 (12.0%)	9 (6.7%)	0.24	36 (6.0)
Co-Morbidities					
	**Diabetes**	9 (18.0%)	29 (21.3%)	0.62	40 (9.0%)^×^
	**Hypertension**	16 (32.0%)	113 (83.1%)	<0.01	133 (30.0%)^×^
	**Obesity**	11 (22.0%)	41 (30.2%)	0.27	124 (27.9%)^×^

^±^ P-value comparing differences by CKD status using either a χ^2^ or Fisher’s exact test

^#^ Other ethnicities includes Maasai, Luguru, Kilindi, Kurya, Mziguwa, Mnyisanzu, Rangi, Jita, Nyambo, and Kaguru

^†^ Professional included any salaried position (e.g. nurse, teacher, government employee, etc.) and retired persons

^‡^ Heart Disease included coronary disease, heart failure, or structural diseases× Proportion from among those tested for these conditions (n = 444)

Among those screened for CKD, diabetes, hypertension, and HIV men (p<0.01) and young adults 18–39 years old (p<0.01) were more likely to be non-responders, and our study sample had a slightly higher prevalence of participants who had obtained a secondary or post-secondary education (20.8%) when compared to the regional average (14.6%; p = 0.03). Among those who were screened for disease, there was no significant difference in occupation (p = 0.70) or ethnicity (p = 0.88) between the responders and non-responders. The most common reasons for non-response in this group were refusal and lost to contact.

### Knowledge

The overall weighted mean knowledge score was 3.28 (95% CI 2.94, 3.63) out of ten possible points (Table 2). Men had a higher mean score compared to women; participants 18–39 years old and 40–59 years old had higher mean scores compared to participants ≥60 years old; Sambaa participants had a lower mean score compared to Chagga and Pare participants; urban residents had a higher mean score compared to rural residents; and participants who were unemployed or professionally employed had higher mean scores compared to farmers/daily wage-earners and small business/vendors. Participants with either a secondary or post-secondary school education had higher mean scores than those with a primary school education or no education.

**Table 2:** Weighted mean knowledge score and crude mean differences in weighted knowledge score from separate univariable linear regression models among study participants; N = 606 (CKD-AFRIKA, 2014).

Category	Variable	Mean knowledge score (95% CI)	Crude mean difference (95% CI)
Demographics	**Gender (%)**		
	Male	3.43 (2.74, 4.11)	Reference
	Female	3.18 (2.88, 3.48)	-0.25 (-0.98, 0.49)
	**Age (%)**		
	18–39 years old	3.33 (2.92, 3.73)	0.74 (0.63, 1.12)
	40–59 years old	3.63 (3.32, 3.93)	1.04 (0.68, 1.40)
	60+ years old	2.58 (2.14, 3.03)	Reference
	**Ethnicity (%)**		
	Chagga	3.39 (3.09, 3.70)	Reference
	Pare	3.12 (2.31, 3.95)	-0.25 (-1.16, 0.65)
	Sambaa	2.80 (2.34, 3.27)	-0.58 (-1.07, -0.90)
	Other	3.30 (2.40, 4.21)	-0.08 (-1.13, 0.97)
	**Setting**		
	Rural	2.99 (2.40, 3.58)	Reference
	Urban	3.73 (3.45, 4.02)	0.74 (0.20–1.28)
Socials Characteristics	**Education (%)**		
	None	1.87 (1.19, 2.54)	Reference
	Primary	3.06 (2.63, 3.50)	1.19 (0.70, 1.69)
	Secondary	4.21 (3.51, 4.94)	2.35 (1.30, 3.39)
	Post-Secondary	5.01 (4.06, 5.97)	3.15 (2.04, 4.26)
	**Occupation (%)**		
	Unemployed	4.27 (3.66, 4.88)	1.16 (0.44–1.87)
	Farmer/Wage Earner	3.11 (2.64, 3.59)	Reference
	Small Business/Vendors	3.12 (2.78, 3.47)	0.01 (-0.60, 0.61)
	Professional	3.96 (3.10, 4.81)	0.84 (-0.17, 1.86)
Self-Reported Medical History			
	**No NCD**^‡^	3.22 (2.80, 3.63)	Reference
	**Any NCD**	3.54 (2.96, 4.12)	0.32 (-0.42, 1.07)
	**No HIV/AIDS**	3.27 (2.92, 3.61)	Reference
	**HIV/AIDS**	3.87 (3.22, 4.51)	0.60 (0.01, 1.20)
Overall		3.28 (2.94, 3.63)	—

^‡^ Non-communicable diseases (NCDs) included diabetes, stroke, hypertension, COPD/asthma, kidney disease, or heart disease

In univariable regression, younger age (< 60 years old), living in an urban setting, unemployed occupation, self-reported history of HIV, and higher levels of education were all significantly associated with a higher mean knowledge score (Table 2); however, only education and setting (urban or rural) remained in the final model with level of education having the strongest independent association with mean knowledge score (Table 3). Adjusting for urban status, mean knowledge score increased an average of 70% for each unit increase in level of education with participants with a post-secondary education scoring more than twice as high (MD 2.85; 1.72, 3.99) as those with no formal education. In the same model, urban residents had a higher mean knowledge score (MD 0.47; -0.03, 0.99) compared to rural participants.

**Table 3:** Results of multi-variable linear regression model of weighted mean knowledge score and adjusted mean differences; N = 606 (CKD-AFRIKA, 2014).

Setting	Mean Knowledge Score (95% CI)	Mean Difference(95% CI)*
**Rural**	2.99 (2.40, 3.58)	Reference
**Urban**	3.73 (3.45, 4.02)	0.47 (-0.03, 0.99)
Education (%)		
**None**	1.87 (1.19, 2.54)	Reference
**Primary**	3.06 (2.63, 3.50)	1.17 (0.59, 1.76)
**Secondary**	4.21 (3.51, 4.94)	2.21 (1.20, 3.23)
**Post-Secondary**	5.01 (4.06, 5.97)	2.85 (1.72, 3.99)
Overall	3.28 (2.94, 3.63)	—

*Adjusted for setting (urban or rural) and level of education

Overall, participants scored the highest on the items pertaining to the normal function of the kidney (Q7, Q8) and the diagnosis and prevention of kidney disease (Q5, Q6), and they scored the lowest on the questions pertaining to the etiology (Q1–3) and treatment of kidney disease (Q9, Q10) (Table 4).

**Table 4:** Prevalence of Knowledge and Attitudes, stratified by CKD and CKD risk status.

Survey Item	Correct (%; 95% CI)		
	Overall (n = 606)	With CKD (n = 50)	At Risk for CKD (n = 136)
Knowledge Domain (% correct; 95% CI)			
**Etiology**			
Q1 (do you think high blood pressure can cause kidney disease?)	17% (13.5,20.0)	25% (9.2, 52.5)	17% (11.2, 24.9)
Q2 (do you think high blood sugar (diabetes) can cause kidney disease?)	26% (21.0, 30.9)	29% (15.0, 49.6)	24% (16.5, 33.8)
Q3 (drinking alcohol can cause kidney disease?)	11% (7.2, 16.9)	8% (3.0, 18.1)	10% (4.6, 21.7)
**Diagnosis**			
Q4 (a person can tell if he/she has kidney disease just by the color, quality, or smell of urine?)	27% (21.5, 32.7)	39% (19.5, 63.3)	22% (14.1, 33.9)
Q5 (kidney disease can only be diagnosed by a test at the hospital?)	54% (46.0, 62.0)	58% (37.7, 75.9)	57% (45.1, 67.4)
Q6 (kidney disease can be prevented if you follow the advice of a medical doctor?)	78% (66.3, 85.8)	85% (63.9, 94.4)	75% (6.0, 85.0)
**Normal Function**			
Q7 (do the kidney control body temperature…?)	25% (19.4, 31.1)	32% (13.0, 58.8)	25% (17.2, 33.8)
Q8 (the kidneys filter waste products from the blood?)	52% (44.3, 59.8)	69% (43.2, 86.2)	46% (38.9, 53.0)
**Treatment**			
Q9 (dialysis is a form of treatment for kidney disease?)	14% (9.9, 19.0)	5% (2.1, 10.6)	11% (6.7, 17.0)
Q10 (antibiotics are a form of treatment for kidney disease?)	26% (20.4, 32.3)	36% (17.2, 59.7)	25% (17.5, 33.7)
**Mean Knowledge Score (95% CI)**	3.28 (2.94, 3.63)	3.85 (3.03, 4.66)	3.04 (2.17, 3.90)
Attitudes Domain (% Positive Response; 95% CI)			
**Learning and Health Concerns**			
Q1 (have you thought you may have kidney problems?)	25% (18.5, 34.1)	36% (17.8, 58.4)	34% (21.2, 49.2)
Q2 (do you like the idea of learning about kidney problems?)	97% (94.8, 98.3)	98% (89.5, 99.7)	97% (90.6, 98.8)
Q3 (…would you be worried about your future?)	23% (18.9, 28.8)	46% (25.9, 68.0)	15% (7.53, 26.8)
Q6 (…would you be worried about your chances of survival?)	27% (20.2, 36.0)	39% (16.7, 67.5)	18% (9.32, 30.5)
**Economic Concerns**			
Q5 (…would you be worried about your ability to work?)	30% (23.2, 37.1)	40% (20.0, 64.4)	19% (10.3, 32.6)
Q8 (do you think the cost of kidney disease would be a problem for you?)	73% (68.2, 77.5)	86% (71.5, 93.6)	73% (61.3, 81.8)
**Social Impact**			
Q4 (…would you be worried about your reputation in the community?)	25% (18.6, 33.6)	29% (12.7, 53.8)	14% (7.68, 23.2)
Q7 (do you think kidney disease is a problem in the Region?)	47% (25.9, 69.1)	51% (40.3, 62.1)	49% (42.0, 56.6)

### Attitudes

Participants showed a strong interest in learning more about kidney disease as well as a frequent concern about the disease’s health, economic, and social impact (Table 4). Nearly all participants (97.0%; 95% CI 94.8, 98.3%) were interested in learning more about kidney disease, and a quarter of participants (25.5%; 95% CI 18.5, 34.1%) had personally worried about their own kidney health. There was no significant difference by demographics, social characteristics, or self-reported medical history in those who reported that they had worried about their kidney health.

The health impact of a diagnosis of kidney disease was a concern for many participants. More than a quarter (27.3%; 95% CI 20.2, 36.0%) of participants were worried about their chances of survival if they were to receive a diagnosis of kidney disease, and the economic impact of a diagnosis of kidney disease was an even greater concern. Nearly a third of participants (30.0%; 95% CI 23.3, 37.1%) were worried about their ability to work if they were to receive a diagnosis of kidney disease and almost three-fourths (73.1%; 95% CI 68.2, 77.5%) worried about the cost of kidney disease irrespective of their occupation (p = 0.63) or education level (p = 0.11).

Participants were also worried about the social and community impact of kidney disease. Half the participants (49.3%; 95% CI 42.0, 56.6%) reported that they believed kidney disease to be a problem within the Kilimanjaro region and a quarter (25.4%; 95% CI 18.6, 33.6%) were worried about their reputations within the community if they were to receive a diagnosis of kidney disease.

### Practices

Overall, participants were willing to seek healthcare from multiple sources for kidney disease. The vast majority responded that they would be likely or very likely to seek care from a biomedical clinic (97.8%; 95% CI 95.6, 98.9%), but many also reported that they would still be likely or very likely to seek advice from a traditional healer (15.2%; 95% CI 9.1, 24.5%) or would be likely or very likely to use traditional medicines for the treatment of kidney disease (33.8%; 95% CI 25.0, 43.9%). Some also reported that they would be likely or very likely to seek self-treatment with home remedies (14.0%; 95% CI 9.1, 20.6%). These modes of healthcare access did not vary significantly by age, gender, urban/rural setting, education, or occupation (all p values >0.25).

Participants were also willing to engage with technology in their healthcare. Some participants were willing to be contacted by email (14.0%; 95% CI 9.2, 20.4%) regarding care for kidney disease. This willingness to be contacted varied significantly by male gender compared to female gender (MD 0.15; 0.06, 0.23), living in an urban setting compared to a rural setting (MD 0.18; 95% CI 0.09, 0.27), and achievement of a secondary education or greater compared to a primary education or less (MD 0.26; 95% CI 0.11, 0.41). Most participants were willing to be contacted by cell phone (94.3%; 95% CI 90.1, 96.8%) regarding their care which did not vary significantly by age, gender, education, or urban/rural setting (all p values >0.30).

### Knowledge, Attitudes, and Practices among those with CKD or at risk for CKD

From the 606 enrolled participants, 444 completed a validated KAP survey and were tested for kidney-related conditions including CKD, diabetes, hypertension, and HIV (Fig 1). Among these participants, 50 (11.3%) had CKD, 136 (30.6%) had no evidence of CKD but were considered to be at high risk for it, and 258 (58.1%) had no CKD and were considered to be at low risk for it.

Most of the participants with CKD were urban residents (n = 47; 94%), female (n = 33; 66%), ethnically Chagga (n = 36; 72%), had a primary school level of education (n = 34; 68%) and worked in a self-employed small business/vendor (n = 19; 38%). The mean age of CKD participants was 49.6 years (SD 16.3). Few participants with CKD were aware of having it (n = 5; 10%).

Among the participants at risk for CKD, 113 (83.0%) had poorly controlled hypertension, 29 (21.3%) had poorly controlled diabetes, and 7 (6.2%) had poorly controlled HIV. Similar to those participants with CKD, these at-risk participants were also mostly female (n = 93; 69%), ethnically Chagga (n = 95; 69.9%), and had only a primary school level of education (n = 92; 67.7%); however, they were more likely to be rural (n = 38; 27.9%) compared to those with CKD (p<0.01).

The overall mean knowledge score was 3.85 (95% CI 3.03, 4.66) among participants with CKD and 3.04 (95% CI 2.17, 3.90) among participants at high risk for CKD (Table 4). Participants with CKD scored slightly higher than those who did not have CKD but were at risk for it (MD 0.81; 95% CI -2.04, 0.42) as well as the general population (MD 0.56; -0.36, 1.48). Participants with CKD (n = 50) had knowledge about the normal, filtering function of the kidneys (69.0%; 95% CI 43.2, 86.2) and the ability of biomedical care to prevent kidney disease (85.2%; 95% CI 63.9, 94.4), but they had very limited knowledge about the etiologies, symptoms, and treatments for kidney disease.

These participants had a high interest in learning more about kidney disease (98.2%; 95% CI 89.4, 99.7), but despite having CKD themselves, only a third (35.5%; 95% CI 17.8, 58.4) had ever thought or worried that they had kidney problems (Table 4). Notwithstanding their low awareness, many participants with CKD were concerned about the health and economic impact of a diagnosis of kidney disease. Over a third (39.5%; 95% CI 16.7, 67.5) of participants were worried about their chances of survival if they were to receive a diagnosis of kidney disease, and many participants (40.2%; 95% CI 20.0, 64.4) were worried about their ability to work if they were to receive a diagnosis of kidney disease. Costs for CKD care were also a concern for more than two-thirds (85.8%; 95% CI 71.5, 95.6) of participants with CKD.

Participants with CKD were willing to seek healthcare from many sources. All (100%) were willing to seek healthcare from a biomedical clinic, but some also reported that they were willing to seek healthcare from traditional healers (12.0%; 95% CI 2.70, 36.9), self-treatment with home remedies (28.8%; 95% CI 13.2, 51.7), or use traditional medicines in the treatment of kidney disease (39.5%; 19.4, 63.8). Likewise, participants at high risk for CKD also were willing to seek healthcare from many sources including traditional healers (14.6%; 95% CI 5.9, 31.9), self-treatment with home remedies (17.7%; 95% CI 9.4, 31.0), or use traditional medicines in the treatment of kidney disease (34.0%; 95% CI 21.2, 49.6).

## Discussion

Community-based adults in northern Tanzania have limited knowledge pertaining to the kidneys and kidney disease. Although overall knowledge and knowledge specific to the etiologies, symptoms, and treatments of kidney disease was low, participants knew modest amounts about the normal function of the kidneys as filtering organs as well as the role of biomedical care in the diagnosis and prevention of kidney disease. This baseline knowledge may serve as a solid foundation upon which to educate further and enhance understanding, especially in the context of favorable attitudes toward learning more about kidney disease. Improving health outcomes for CKD through targeted educational interventions in high risk populations may be integral for improving NCD outcomes more broadly; however, such CKD health programs will need to be sensitive to healthcare practice preferences that include both biomedical and traditional medicines.

In our study population, education and setting (urban/rural) had the strongest associations with mean knowledge score for kidney disease. In high-income countries, level of education is directly associated with better health outcomes in patients with kidney disease, and education can moderate the effectiveness of interventions [10]. Health literacy, which is the ability to obtain, process, and understand basic health information in order to make healthcare decisions, may be one means by which education is associated with health outcomes, and in high-income settings, targeting individuals at high risk for low health literacy has been effective in improving CKD care [11, 12]. In northern Tanzania, targeting rural populations who have limited healthcare access and persons with low levels of education who are at high risk of low health literacy, may allow for more efficient interventions that better account for these disparities.

We identified attitudes that were characterized by a strong interest in learning about kidney disease, and these participants also had frequent concerns about the economic, health, and social impact of being diagnosed with CKD. Public health efforts aimed at improving awareness and reducing these concerns have been effective in other low-resource settings outside of sub-Saharan Africa, and our findings suggest that such efforts would also be well-received in northern Tanzania [13–15]. Use of technology-based platforms may be effective in implementing such programs in this setting where mobile health (mhealth) technologies have been used successfully in public health efforts related to maternal-child health, tuberculosis, and HIV [16, 17]. Although the use of technology-based platforms for improving NCD care in sub-Saharan Africa is not well established, our assessment of healthcare practices related to kidney disease suggested that mobile phones would be a potentially well-received means of facilitating such CKD public health efforts, and in local urban settings, email may also be useful for health communication and education among certain populations [18].

Regardless of CKD status, participants were willing to seek healthcare from a variety of sources that included traditional healers and traditional medicine use. This is consistent with published literature showing that traditional health services are accessed frequently for chronic diseases, and that their use is not limited to rural, low-income, or elderly populations [19]. However, though traditional medicines can be an effective form of treatment for many conditions, some are also known to be nephrotoxic—even ones that are used specifically in the local treatment of kidney disease [20–27]. As such, increasing regional awareness about CKD or even acute kidney injury may have unintended consequences if not fully informed and sensitive to traditional practices that may include the use of traditional medicines with adverse effects on the kidney.

Our study has many strengths. To our knowledge, this is the first community-based assessment of knowledge, attitudes, and practices related to kidney disease in a Swahili-speaking population or in all of East Africa. It is also the first assessment of knowledge, attitudes, and practices related to kidney disease among an East African community-based sample of participants with CKD or at risk for CKD, and our findings will be useful in formulating local, community-based programs. For example, the Nephrology Society of Tanzania (NESOT) operates locally to promote and advance the knowledge and practice of nephrology, and our findings are synergistic with these efforts. Additionally, our survey instrument was tested for construct validity, content validity, and reliability [5]. Finally, because of our sampling methods, these results may also be externally generalizable across the regional and national populations [6].

We also noted a few limitations in our study. As this was a cross-sectional study, causal inferences cannot be drawn and associations may be influenced by confounding from unmeasured variables. Studies in other African populations suggest that the Chronic Kidney Disease Epidemiology Collaboration formula may better at estimating GFR [9, 28]. We chose to use the MDRD formula due to local considerations in medical practices; yet, because there is no validated creatinine-based measurement for GFR in our study population, measurement biases may exist around cut-off values. Furthermore, our study may be subject to non-response bias which can be introduced when the response rate is low and/or when there is a substantial difference between responders and non-responders [29]. To reduce non-response bias we attempted a minimum of two additional off-hour visits, and to address any non-response bias that may have arisen from differences between the respondents and non-respondents, we used sample-balanced weights for age and gender. Our study may also have been subject to reporting and recall bias. Reporting bias can be introduced when participants are reluctant to answer truthfully or are more likely to report seemingly desirable information, and recall bias can be present when participants unknowingly answer incorrectly due to inaccuracies in memory or cultural frame-shifting. To reduce these biases, we used only local native surveyors who spoke Swahili as their first language, conducted the interviews in private when possible, and pre-tested the survey instrument for design flaws as part of the validation process.

In conclusion, in a community-based sample of adults in northern Tanzania we observed low knowledge about kidney disease which was strongly associated with level of education and living in a rural setting. There was a modest knowledge base about the kidneys that could serve as an important foundation upon which to build CKD educational programs to expand awareness and understanding. Targeted efforts would likely be well-received based upon our assessment of local attitudes, and further research is needed to examine the potential use of mobile phones for facilitating CKD education and care. Adults in northern Tanzania, including those with CKD and at risk for CKD, meet their healthcare needs by accessing a variety of sources including traditional healers, and CKD management programs must recognize these important preferences in order to be successful.

## Supporting Information

S1 Appendix KAP Survey Instrument (English and Swahili). (DOCX)
